# Sugammadex-Associated Hypotension, Bradycardia, Asystole, and Death

**DOI:** 10.1155/2020/8767195

**Published:** 2020-06-10

**Authors:** Kazim Mirza, Kathryn Landoski, Dilip Thakar, Jagtar Heir-Singh, Timothy Jackson, Cynthia Kassab

**Affiliations:** MD Anderson Cancer Center, Houston, TX, USA

## Abstract

On December 16, 2015, the Food and Drug Administration (FDA) in the United States approved sugammadex (Bridion, Merck and Co), a modified gamma-cyclodextrin, to be used as a reversal agent. It is a first and unique selective nondepolarizing steroidal muscle relaxant (NDSMR) binding agent with a great affinity for rocuronium and vecuronium. However, there have been several recently published case reports of bradycardia and asystole immediately after sugammadex administration for the reversal. This report presents a case of sugammadex administration followed by rapidly progressing bradycardia leading to asystole and subsequent death. The family has provided the written consent to share this case report.

## 1. Case Report

An 82-year-old patient was admitted for an elective exploratory celiotomy and possible lysis of adhesions due to postoperative chronic bowel obstruction. Six months prior, the patient underwent cystoprostatectomy with bilateral lymph node dissection and creation of an ileal conduit for combined prostate and bladder cancer. In the postoperative course of the cystoprostatectomy, the patient suffered multiple bowel obstructions necessitating a venting g-tube placement and total parenteral nutrition (TPN), which resulted in a major weight loss of 48 pounds in less than a year. His medical history is significant for chronic obstructive pulmonary disease (COPD), hypertension, and dyslipidemia, and he is a smoker of 100 packs per year, currently smoking half a pack per day. His surgical history is significant for right thoracotomy, upper lobectomy, and mediastinal lymph node resection in 2000 for lung cancer, re-operated with right lobectomy, middle lobe wedge resection, and excision of the 5^th^ rib fourteen years later, followed by external beam radiation of a left upper lobe lung cancer in 2018.

Upon the preoperative evaluation for the exploratory laparotomy, his labs were essentially within normal limits, except for a slightly elevated BUN of 32, creatinine of 1.06, considered normal when adjusted to his age, and bilirubin of 0.5 (normal <0.3). A preoperative electrocardiogram (ECG) showed sinus rhythm at 67 bpm, borderline ST elevation, anterior leads, minimal ST depression, and an anterior ECG two months ago with multiple ventricular premature complexes and left ventricular hypertrophy. His American Society of Anesthesiologists (ASA) score was 4 and his ARISCAT score was 47, which combined with his comorbidities implied a high risk for surgical procedures. The patient expressed a will to undergo the surgery because he was experiencing abdominal cramping from the TPN and wanted to be able to eat again. He was home medicated with Azelastine 0.15% nasal spray; hydromorphone 2 mg tab prn and Trelegy 100-62.5-25 mcg daily; and montelukast 10 mg.

On the day of surgery, his right peripherally inserted central catheter (PICC) line was flushed and connected to PlasmaLyte. After all the preoperative checklist had been completed, he was medicated with fentanyl 50 mcg and famidodine 20 md IV and taken to the OR. The usual monitors were placed, and slow intravenous (IV) induction occurred with 2% lidocaine 5 ml, propofol 50 mg, rocuronium 50 mg, and fentanyl 50 mcg. He was subsequently intubated with a 8.0 ETT and placed on mechanical ventilation after breath sounds were assessed at a tidal volume (TV) of 450, rate of 10, inspiratory : expiratory (I : E) ratio of 1 : 2.5, and positive end expiratory pressure (PEEP) of 5 at 50% oxygen. A 20-gauge left radial arterial line and 16-gauge left antecubital fossa IV were also placed. Cefoxitin 2 grams was given for antibiotic prophylaxis, and ertapenem 1 gram was added later to expand the bacterial coverage. The patient was maintained on 4–7% desflurane, and another 100 mcg fentanyl and 1.5 mg hydromorphone given for pain relief throughout the case, which lasted almost 3 hours 40  minutes. A total of 30 mg ephedrine and 150 mcg phenylephrine were given to keep the systolic pressure 100 mmHg and above (his preoperative pressures were 115/64–135/67). Esmolol in 20 mg increments was also given to treat occasion ST segment depression over baseline, with return to baseline levels. A nasal gastric tube was placed at 10:15 at the surgeon's request due to bowel dilatation and placed to low suction after placement was confirmed by the surgeon. A total of another 50 mg rocuronium in divided doses was given to facilitate abdominal resection. At about 11 : 31, a combination of 0.25% bupivacaine and Exparil was infiltrated into the abdominal incision as closure was continuing. Another episode of ST segment depression occurred at about 11:56, so another 10 mg of esmolol times 2 and 50 mcg phenylephrine were given. At 11:58 surgery finished, sugammadex 200 mg (2.9 mg/kg) was given since he had ¼ twitches on the PNS. Shortly after sugammadex administration, he developed rapidly progressing bradycardia resulting in asystole, and 0.2 mg glycopyrolate times 2 was given, followed by 10 mg ephedrine. Subsequently, PEA was noted and CPR started with chest compressions and a code called. The code lasted a total of 1 hour 50 minutes. Multiple doses of epinephrine were given, twice he eventually converted to ventricular rhythms that permitted defibrillation, but he was unable to sustain a blood pressure despite a norepinephrine infusion. Finally, the code was called at 13: 50. On December 16, 2015, the Food and Drug Administration (FDA) in the United States approved sugammadex (Bridion, Merck & Co), a modified gamma-cyclodextrin, to be used as a reversal agent. It is the first and unique selective nondepolarizing steroidal muscle relaxant (NDSMR) binding agent with a great affinity for rocuronium and vecuronium. Despite these patient's comorbidities, there have been a significant number of cases of arrhythmias [1, 2], anaphylaxis, and death directly related to sugammadex administration, which merits further investigation and studies in order to optimize its use [3–5].
